# Developing a dichlorosalicylaldehyde-derived fluorescent probe for monitoring glutathione in a cellular pulmonary ventilation model

**DOI:** 10.3389/fchem.2025.1698116

**Published:** 2025-09-30

**Authors:** Xiao Zhou, Lei Zhang, Yuwen Lao, Bin Zhou, Zhongquan Zhu

**Affiliations:** Department of Anesthesiology, Affiliated Jinhua Hospital, Zhejiang University School of Medicine, Jinhua, China

**Keywords:** fluorescence probe, glutathione level, pulmonary ventilation, cellular model, dichlorosalicylaldehyde derivative

## Abstract

Herein, a fluorescent probe, DSNBD-GSH, was developed from dichlorosalicylaldehyde to monitor glutathione (GSH) in a cellular pulmonary ventilation model. Both the normoxia and hypoxia conditions were evaluated. DSNBD-GSH responded to GSH in a dosage-dependent manner with a fluorescence signal at 478 nm under the excitation of 365 nm. The solution tests indicated that DSNBD-GSH had relatively high sensitivity for GSH, and the photophysical properties were stable in various conditions. Other advantages included rapid response, high selectivity, and low cytotoxicity. Most significantly, monitoring the GSH level in both hypoxia and recovery status allows for visualization of ventilation-related redox changes. This work highlights a referenceable case for improving the pulmonary ventilation approach during the perioperative period.

## 1 Introduction

In modern clinical trials, the perioperative period has been a research hotspot for guaranteeing the health of the patients, while the corresponding anesthesia processes require advanced knowledge of the pulmonary ventilation conditions (Mo et al., 2024; Xu et al., 2024). The pulmonary ventilation regulation is tightly associated with risks such as ischemia-reperfusion injury, oxidative stress, and inflammatory response (Buonanno et al., 2023; Gao et al., 2024; Liu et al., 2022). In chronic obstructive pulmonary disease (COPD) cases, typically, the tidal volume range and positive end expiratory pressure should be ensured in a suitable range (Abualhamael et al., 2024). Commonly, the rational use of anesthetics (such as propofol, sevoflurane, and dexmedetomidine) has been introduced for better regulation of the pulmonary ventilation conditions (Bai et al., 2022; Kawakami et al., 2024; Wang et al., 2024). Accordingly, a lack of enough indicators for precise regulation has become a main limiting factor in the medicinal processes. At present, the main indicators remain physical, including resting ventilation volume, alveolar ventilation volume, maximum ventilation volume, and time lung capacity (Lorenzo-Capella et al., 2025; Miura et al., 2020; Ovenholm et al., 2024). However, physical indicators cannot fulfill the requirements of mechanism investigations and *in-situ* monitoring procedures. Therefore, studying the molecular indicators is an essential task before revealing further interaction networks and developing potential therapeutic approaches (Lee et al., 2020; Zheng et al., 2021).

Distinguished from the hepatic and nephric cases, the pulmonary ventilation models ask for consideration of the oxygen supply variation (Marquis et al., 2021; Wohlrab et al., 2021). In particular, both the normoxia and hypoxia micro-environments should be checked when seeking the possible indicators (Chao et al., 2021). The directly identified molecular indicators involved carbon dioxide (CO_2_) and peroxynitrite (ONOO^−^), which were associated with the reactive oxygen species (ROS) and reactive nitrogen species (RNS) (Barrett et al., 2022; Martinez-Caro et al., 2015). Moreover, tracking the metabolism of lipids and proteins has brought the analysis to the enzyme-related indicators such as carboxylesterase (CE) and cysteine (Cys) (Erdil et al., 2016; Wu et al., 2024). Typically, the nodes in multiple pathways exhibit more information to reveal the interaction mechanisms. Glutathione (GSH), as a key representative, covers the regulation of both the amino acid conversion and sulfur metabolism (Ferreira et al., 2023; Gopika et al., 2024). In combination with other indicators such as C-reactive protein and IL-10, GSH may contribute to describing the pathological status in the pulmonary ventilation processes. One specific sample was the cooperation with the iron ion pool in ferroptosis. The key nodes, including GSH, might reveal the facts in the complex interaction network.

For monitoring GSH, the present methods mainly involve colorimetry (Chen et al., 2025), high-performance liquid chromatography (Kubat et al., 2024), and capillary electrophoresis (Ivanov et al., 2022). Since the pulmonary ventilation processes require *in-situ* potential and good biocompatibility, the above methods cannot fulfill the specific scenario. Recent investigations into the fluorescent probes have met the requirements, which have also facilitated real-case monitoring of GSH (Li et al., 2023; Liu et al., 2025; Long M. et al., 2025; Long Z. Z. et al., 2025; Lu et al., 2025; Wang et al., 2025; Xie et al., 2025; Yan et al., 2025; Zhang et al., 2023). On the basis of the reaction mechanisms, including nucleophilic substitution, Michael addition, and cyclization-departure, the developed probes have realized the selective recognition of GSH from other biological thiols such as Cys and homocysteine (Hcy). In particular, 7-nitrobenzofurazan (NBD) has been regarded as a recognition group with a high success rate (Slowinski et al., 2022). Both the fluorophores and recognition groups affect the photophysical properties of the probes. Thus, to monitor GSH in cellular pulmonary ventilation, novel fluorescent probes with corresponding methodologies are still attractive for the investigators in chemical biology.

**FIGURE 1:**
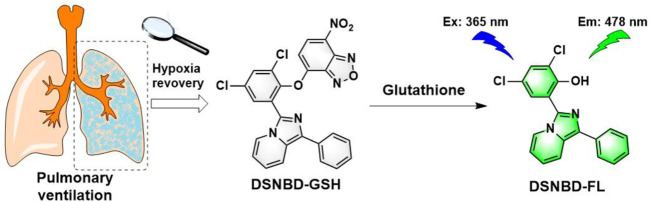
The illustration of the probe DSNBD-GSH for monitoring GSH in the cellular pulmonary ventilation model.

Herein, a dichlorosalicylaldehyde-derived fluorescent probe for monitoring glutathione in the cellular pulmonary ventilation model was prepared and tested (Figure 1). The fluorophore was derived from the original dichlorosalicylaldehyde subunit with the hydroxyl as the electron-donating site and chlorine substitutes as the conformation regulator. The selection of the dichlorosalicylaldehyde source was based on introducing the steric hindrance and reducing the local electron density, which were beneficial for improving the selectivity and photophysical properties. The nitrogen-containing aromatic rings were introduced to form the electron-withdrawing subunit. The structural features of the fluorophore indicated the basic mechanism of the fluorescence generation as excited-state intramolecular proton transfer (ESIPT) according to some previous reports (Padalkar and Seki, 2016; Sedgwick et al., 2018; Zhou and Han, 2018). By anchoring the NBD recognition group, the probe DSNBD-GSH was acquired. The experiments on the photophysical properties were carried out according to convention; both the normoxia and hypoxia conditions were considered. The cellular pulmonary ventilation model was constructed, and the imaging performance of the probe was thereby studied. DSNBD-GSH was expected to serve the imaging scenario in living pulmonary cells.

## 2 Experimental

### 2.1 Materials and methods

The chemicals and agents were purchased from Energy Chemical Co. Ltd. (Shanghai, China). The cell lines were acquired from American Type Culture Collection (ATCC) and stored in Zhejiang University School of Medicine. The nuclear magnetic resonance (NMR) spectra were recorded on a DRX-600 spectrometer (Bruker, Karlsruhe, Germany). The high-resolution mass spectra (HRMS) were recorded on a 6475 triple quadrupole lipid chromatography-mass spectrometry workstation (Agilent, Santa Clara, United States). The UV-vis measurement was performed on a UV2450 spectrometer (Shimazu, Kyoto, Japan). The fluorescence measurement was conducted on an F-7000 fluorescence spectrophotometer (Hitachi, Tokyo, Japan). The intracellular imaging was carried out with an FV-1000 confocal laser scanning biological microscope (Olympus, Tokyo, Japan).

The stock solution of the probe DSNBD-GSH was set at 1.0 mM in dimethyl sulfoxide (DMSO), and the other concentrations were acquired by dilution with phosphate buffer saline (PBS) and pure water. The solution pulmonary ventilation model was constructed by bubbling in 90 mL CO_2_ with rapid shaking for 2 s as described in the previous literature (Zhu et al., 2024). The resulting model was close to the 45 mmHg PaCO_2_ hypercapnia condition with a common alveolar ventilation (VA) as 5 L/min. The main conditions included photomultiplier voltage 600 V, excitation and emission slit widths 5 nm * 5 nm, pH 7.4, 37 °C, and 20 min incubation under an excitation of 365 nm. The cellular pulmonary ventilation model was constructed by setting the culture environment of the atmosphere proportion to mimic the hypoxia and hypoxia recovery groups. The signals in the green channel of 450–600 nm were collected.

### 2.2 Fluorescence quantum yields determination

The fluorescence quantum yields (FQYs) determination relied on conversion with the rhodamine B ethanol solution (0.69 under the excitation of 365 nm) following a typical reference method. Accordingly, the FQY value of the DSNBD-GSH probe was 0.11, while that of the detecting product DSNBD-FL was 0.69.

### 2.3 Limit of detection determination

The limit of detection (LOD) determination relied on the formula LOD = 3.29σ/k, where 3.29 was the International Union of Pure and Applied Chemistry (IUPAC) set coefficient; σ was the background standard deviation from 25 independent measurements; and k was the slope value of the linear correlation. Accordingly, σ = 0.2962, k = 1.244, and LOD = 0.78 µM.

### 2.4 Cell culturing and intracellular imaging

Human non-small cell lung cancer cells (A549 cell line) and normal lung bronchial epithelial cells (BEAS-2B cell line) were selected and cultured in Dulbecco’s Modified Eagle’s Medium (DMEM) with 10% fetal bovine serum (FBS) and 1% penicillin-streptomycin. The condition was set to the following: 5% CO_2_-containing atmosphere, 37 °C, and 24 h. The thiazole blue (MTT) assay was conducted to check the cytotoxicity by recording the optical density values at 570 nm.

Then, A549 cells were selected for intracellular imaging. They were divided into five groups based on distinct culturing and incubation conditions. The normoxia atmosphere (20% O_2_ and 5% CO_2_) was used for the initial three groups, while the hypoxia condition (5% O_2_, 50% CO_2_, and N_2_ supplied) was used for the last two groups. The first group was incubated with PBS for 30 min and DSNBD-GSH (10 μM) for 30 min and imaged. The second group was incubated with *N*-Ethylmaleimide (NEM, a sulfite scavenger, 1 mM) for 30 min, to eliminate the biological thiols, and DSNBD-GSH (10 μM) for 30 min and imaged. The third group was incubated with *N*-Ethylmaleimide (NEM, a sulfite scavenger, 1 mM) for 30 min, then *N*-acetyl-L-cysteine (NAC, 1 mM) for 30 min to supply the endogenous generation of GSH, and DSNBD-GSH (10 μM) for 30 min and imaged. The fourth group, pre-cultured in hypoxia, was incubated with PBS for 30 min and DSNBD-GSH (10 μM) for 30 min and imaged. The fifth group, pre-cultured in hypoxia, was incubated with 30% H_2_O_2_ in the last 1 h to mimic a quick hypoxia recovery, then incubated with PBS for 30 min and DSNBD-GSH (10 μM) for 30 min and imaged. The quick hypoxia recovery was controllable on living cells with the limited oxidative stress (Mailloux, 2015; Yang et al., 2024). Thus, the normoxia, hypoxia, and hypoxia recovery status were all covered.

### 2.5 Chemical synthesis

**FIGURE 2:**
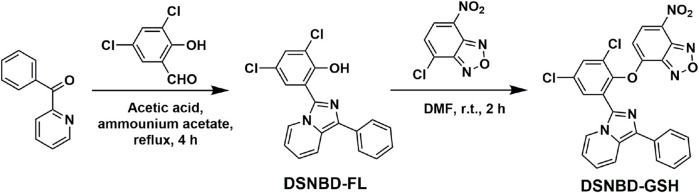
The general synthetic route of the probe DSNBD-GSH from dichlorosalicylaldehyde.

The general synthetic route of the probe DSNBD-GSH is shown in Figure 2. The initial reaction was the formation of the fluorophore. The raw material 3,5-dichlorosalicylaldehyde (0.29 g, 1.5 mmol) was dissolved in 15 mL acetic acid in a round bottom flask of 50 mL. Then, phenyl(pyridin-2-yl)methanone (0.27 g, 1.5 mmol) and ammonium acetate (0.15 g, 2 mmol) were added. The mixture was stirred in reflux continuously for 4 h. The completion of the reaction was confirmed by the thin-layer chromatography (TLC). Then, the pH was adjusted to neutral conditions, and the precipitate was purified through column chromatography with the eluent of petroleum ether and ethyl acetate (4:1). The yellow solid obtained was the fluorophore DSNBD-FL at a yield of 53.8%. The following was calculated for [C_19_H_13_Cl_2_N_2_O]^+^: 355.0405, found: 355.0400: ^1^H NMR (600 MHz, CDCl_3_) *δ* 13.14 (s, 1H), 8.46 (d, *J* = 7.3 Hz, 1H), 7.93 (d, *J* = 9.2 Hz, 1H), 7.85 (d, *J* = 7.4 Hz, 2H), 7.66 (d, *J* = 2.2 Hz, 1H), 7.48 (t, *J* = 7.6 Hz, 2H), 7.39 (d, *J* = 2.2 Hz, 1H), 7.36 (t, *J* = 7.4 Hz, 1H), 6.96 (dd, *J* = 9.1, 6.4 Hz, 1H), 6.83 (t, *J* = 6.8 Hz, 1H). ^13^C NMR (150 MHz, CDCl_3_) *δ* 151.45, 133.60, 132.93, 130.19, 129.56, 128.94, 127.52, 127.51, 126.76, 123.70, 123.57, 122.40, 122.04, 121.23, 119.71, 115.82, 115.25. HRMS (ESI-Q-TOF m/z).

Subsequently, DSNBD-FL (1 mmol) was dissolved in 5 mL *N*,*N*-dimethylformamide (DMF) in a round-bottom flask of 20 mL under nitrogen protection. Then 7-nitrobenzofurazan (0.20 g, 1 mmol) and potassium carbonate (0.14 g, 1 mmol) were added. The mixture was stirred continuously at room temperature for 2 h. The completion of the reaction was confirmed by the TLC. The mixture was filtered and washed with cold methanol. The residue was freeze-dried. The obtained dark yellow solid was the probe DSNBD-GSH at a yield of 61.2%. The following was calculated for [C_25_H_14_Cl_2_N_5_O_4_]^+^: 518.0422, found: 518.0397: ^1^H NMR (600 MHz, CDCl_3_) *δ* 8.21 (d, *J* = 8.3 Hz, 1H), 7.95 (d, *J* = 7.2 Hz, 1H), 7.80 (d, *J* = 2.5 Hz, 1H), 7.68-7.66 (m, 2H), 7.51 (d, *J* = 7.3 Hz, 2H), 7.30 (t, *J* = 7.6 Hz, 2H), 7.21-7.19 (m, 1H), 6.82 (dd, *J* = 8.1, 6.5 Hz, 1H), 6.75 (t, *J* = 7.7 Hz, 1H), 6.41 (d, *J* = 8.2 Hz, 1H). ^13^C NMR (150 MHz, CDCl_3_) *δ* 150.90, 145.38, 144.18, 143.93, 133.97, 132.92, 132.01, 131.57, 131.32, 130.03, 129.17, 128.98, 128.03, 127.68, 126.68, 122.27, 121.74, 119.03, 115.25, 108.80. HRMS (ESI-Q-TOF m/z).

## 3 Results and discussion

### 3.1 Synthesis of the probe DSNBD-GSH

As shown in Figure 2, the probe DSNBD-GSH was synthesized in two main steps. Initially, the conjugated structure of the raw material dichlorosalicylaldehyde was enlarged with the formation of nitrogen-containing aromatic rings to form the fluorophore DSNBD-Fl. Subsequently, the NBD recognition group was added onto the hydroxyl site of the fluorophore to generate the probe DSNBD-GSH. All the synthesized compounds were checked with the NMR and HRMS data (Supplementary Figures S1–S6).

### 3.2 In solution tests on photophysical properties

Herein, as mentioned in the experimental section, the solution pulmonary ventilation model was constructed by bubbling in CO_2,_ and the result was a mimicked hypercapnia-like status with corresponding calculation parameters. Both the normoxia and hypoxia conditions were considered during the preliminary tests on the absorption and fluorescence variation. In normoxia, the UV-vis spectra of the probe DSNBD-GSH (10 μM) exhibited two peaks at 300 nm and 360 nm, while the incubation with GSH (1 mM) caused the decrease of the peak at 360 nm (Supplementary Figure S7A). In hypoxia, the absorption variation was almost the same. When the excitation wavelength was set as 365 nm, in normoxia, no obvious fluorescence peak was observed in the spectrum of the probe DSNBD-GSH (10 μM), while the incubation with GSH (1 mM) led to the appearance of a notable fluorescence peak at 478 nm (Supplementary Figure S7B). In hypoxia, the fluorescence variation was also similar to that in normoxia. Since the solution pulmonary ventilation model did not seriously affect the tests on the photophysical properties, the following experiments used the normoxia condition for conciseness. The FQY value of the probe DSNBD-GSH was 0.11, while that of the detecting product DSNBD-FL was 0.69. The above results suggested that DSNBD-GSH was available for serving a monitoring system with the turning-on fluorescence response.

**FIGURE 3:**
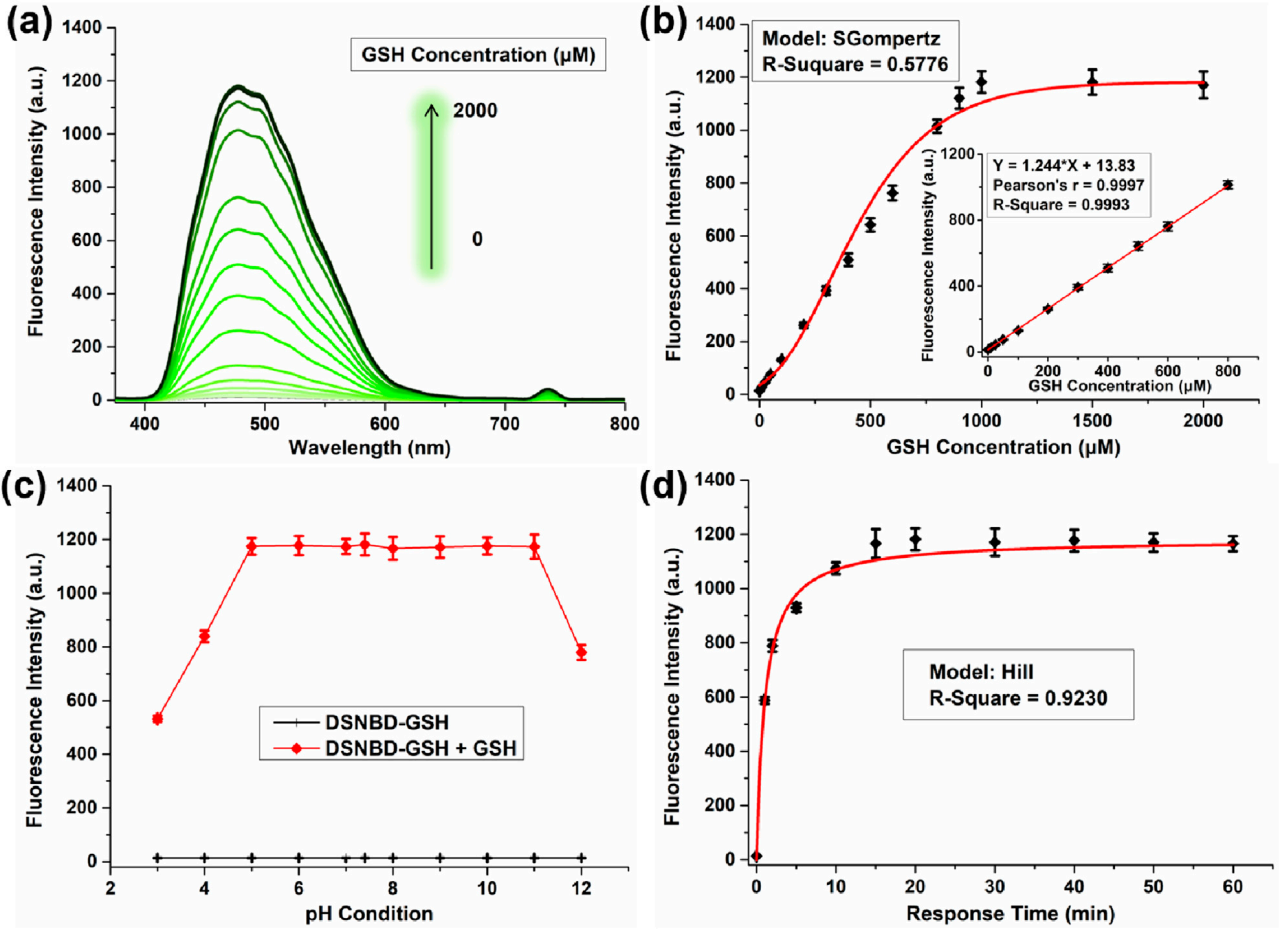
**(a)** The fluorescence spectra of DSNBD-GSH (10 μM) with various concentrations of GSH (0–2000 μM). **(b)** The correlation between the fluorescence intensity at 478 nm and the concentration of GSH (0–2000 μM); Insert: The linear correlation (0–800 μM). **(c)** The fluorescence intensity variation of DSNBD-GSH (10 μM) with GSH (1 mM) in various pH conditions (3.0–12.0). **(d)** The fluorescence intensity variation of DSNBD-GSH (10 μM) with GSH (1 mM) in various incubation times (0–60 min). General testing conditions: 600 V, 5 nm * 5 nm, excitation wavelength 365 nm, pH 7.4, 20 min, and 37 °C.

By convention, the standard curve was constructed to describe the correlation between the fluorescence intensity of the solution containing DSNBD-GSH (10 μM) at 478 nm and the concentration of GSH (0–2000 μM). Along with an increase in the GSH concentration, the fluorescence intensity enhanced to reach a plateau when the GSH concentration was 1 mM (Figures 3a,b). In the range of 0–800 μM, there was a linear correlation with the Pearson’s coefficient of 0.9997 (Figure 3b Insert). The LOD value was calculated as 0.78 µM, which suggested a relatively high sensitivity. Moreover, the monitoring system also required the stability of the reporting signals in various testing conditions. The pH, response time, and incubation temperature were all commonly concerned parameters. For the pH condition, the probe DSNBD-GSH remained almost non-fluorescent in the range of 3.0–12.0, while a certain intensity was steadily observed after the response towards GSH in the range of 5.0–11.0 (Figure 3c). The reporting signal intensity was tightly associated with the pH condition, which was consistent with the ESIPT mechanism. When the pH condition was not ideal, the fluorescence intensity of the detecting product decreased accordingly. For the response time, the reaction between DSNBD-GSH and GSH was absolutely completed within 15 min (Figure 3d). It was a relatively rapid response among the reports for monitoring GSH. For the incubation temperature, the probe DSNBD-GSH remained almost non-fluorescent in the range of 25 °C–45 °C, while a certain intensity was steadily observed after the response towards GSH in the range of 35 °C–45 °C (Supplementary Figure S8). In consideration of all the testing conditions, the solution containing DSNBD-GSH suggested the stable monitoring of GSH, which covered the requirements for the pulmonary ventilation-related conditions.

### 3.3 Selectivity of the probe DSNBD-GSH

**FIGURE 4:**
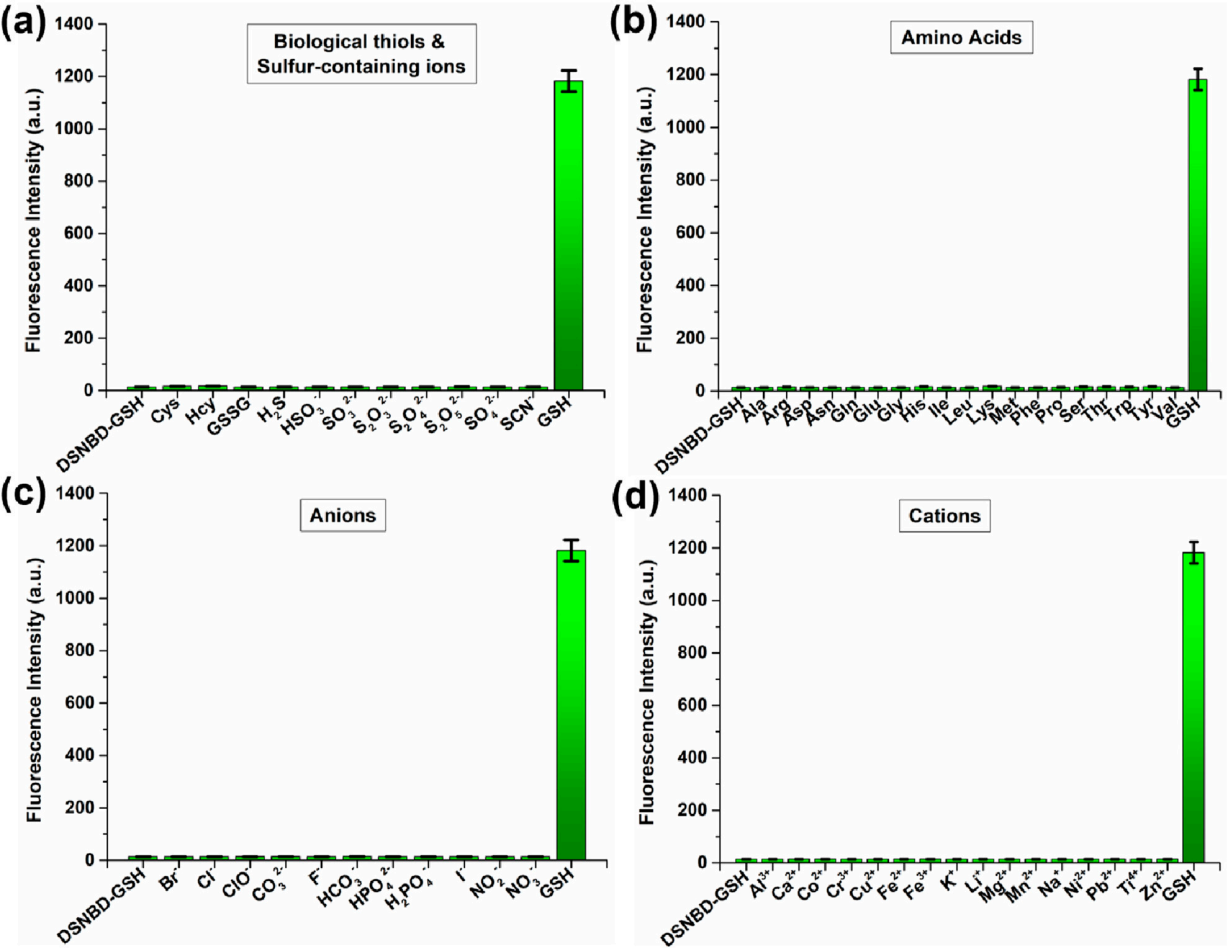
The fluorescence intensity variation of DSNBD-GSH (10 μM) at 478 nm after the incubation with various analytes: **(a)** biological thiols and sulfur-containing ions; **(b)** amino acids; **(c)** anions; and **(d)** cations. General testing conditions: 600 V, 5 nm * 5 nm, excitation wavelength 365 nm, pH 7.4, 20 min, and 37 °C.

The surroundings of the GSH detection require high selectivity, thus the tests on this point should be unfolded for the prepared probe DSNBD-GSH. The concentration of DSNBD-GSH was set as 10 µM, and the concentrations of the analytes were all set as 1 mM. Regarding GSH as the research object, the most significant competitors included other biological thiols as well as sulfur-containing ions. After the comparison with the species, including Cys, Hcy, GSSG, H_2_S, HSO_3_^−^, SO_3_^2-^, S_2_O_3_^2-^, S_2_O_4_^2-^, S_2_O_5_^2-^, SO_4_^2-^, and SCN^−^, it was found that only GSH led to the remarkable fluorescence response at 478 nm (Figure 4a). In particular, the probe DSNBD-GSH might directly distinguish GSH from Cys and Hcy, which was essential in the probes for sulfur metabolism. Other competitors were tested in sequence. The species cover amino acids (Ala, Arg, Asp, Asn, Gln, Glu, Gly, His, Ile, Leu, Lys, Met, Phe, Pro, Ser, Thr, Trp, Tyr, Val) in Figure 4b, anions (Br^−^, Cl^−^, ClO^−^, CO_3_^2-^, F^−^, HCO_3_^−^, HPO_4_^2-^, H_2_PO_4_^−^, I^−^, NO_2_^−^, NO_3_^−^) in Figure 4c, and cations (Al^3+^, Ca^2+^, Co^2+^, Cr^3+^, Cu^2+^, Fe^2+^, Fe^3+^, K^+^, Li^+^, Mg^2+^, Mn^2+^, Na^+^, Ni^2+^, Pb^2+^, Ti^4+^, Zn^2+^) in Figure 4d. No obvious fluorescence response was observed throughout the tests of the above-mentioned competitors. Basically, the selectivity towards GSH relied on the differences in affinity, while the fluorescence generation of ESIPT further enlarged the differences in fluorescence intensity. Therefore, establishing the monitoring system with DSNBD-GSH ensured high selectivity towards GSH.

### 3.4 Imaging GSH in living pulmonary cells

Before the intracellular imaging procedure, the cytotoxicity of the probe DSNBD-GSH on living pulmonary cells (A549 and BEAS-2B cell lines) was investigated with the MTT assay. Along with the increase of the probe concentration to reach 50 μM, the cell viability of both cell lines remained over 90% (Supplementary Figures S9A,B). The low cytotoxicity was suitable for imaging in living pulmonary cells.

**FIGURE 5:**
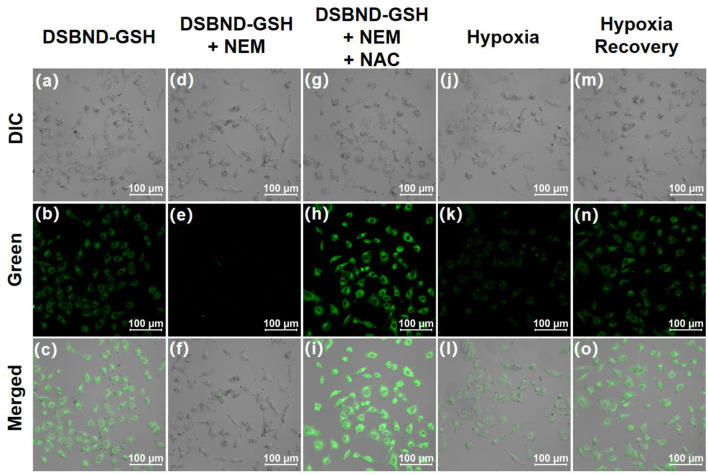
The confocal imaging of living A549 cells in various culturing and incubating conditions before being imaged: **(a–c)** cultured in 20% O_2_, 5% CO_2_, incubated with PBS for 30 min, DSNBD-GSH (10 μM) for 30 min; **(d–f)** cultured in 20% O_2_, 5% CO_2_, incubated with NEM (1 mM) for 30 min, DSNBD-GSH (10 μM) for 30 min; **(g–i)** cultured in 20% O_2_, 5% CO_2_, incubated with NEM (1 mM) for 30 min, NAC (1 mM) for 30 min, DSNBD-GSH (10 μM) for 30 min; **(j–l)** cultured in 5% O_2_, 50% CO_2_, N_2_ supplied, incubated with PBS for 30 min, DSNBD-GSH (10 μM) for 30 min; **(m–o)** cultured in 5% O_2_, 50% CO_2_, N_2_ supplied, incubated with 30% H_2_O_2_ in the last 1 h of culturing, PBS for 30 min, DSNBD-GSH (10 μM) for 30 min. The Differential Interference Contrast (DIC) channel showed the bright field. General testing conditions: excitation wavelength 365 nm, pH 7.4, 37 °C, green channel of 450–600 nm, and scale bar 100 µm.

Afterwards, the A549 cells were divided into five groups for the confocal imaging procedure. The first group was cultured in normoxia before being incubated with PBS and the probe DSNBD-GSH in sequence to serve as the control group (Figures 5a–c). The fluorescence signal with a certain intensity in the green channel was consistent with the existing GSH level in living pulmonary cells. The second group was also cultured in normoxia, and NEM was used to clean up the biological thiols; thus, the following incubation with the probe resulted in the disappearance of the fluorescence signal (Figures 5d–f). The third group was based on the second one. After the elimination by NEM, the subsequent incubation of NAC supplied the endogenous generation of GSH (Figures 5g–i). The living pulmonary cells degraded the acetyl and used Cys to synthesize GSH, which was visualized by the generation of a notable fluorescence signal in the green channel. The fourth group was cultured in hypoxia, incubated with PBS and the probe DSNBD-GSH in sequence before being imaged (Figures 5j–l). Compared with the control status, the hypoxia condition led to a decrease in the fluorescence signal, which reflected the decreased GSH level as a potential biomarker for the unideal pulmonary ventilation. The fifth group was cultured in hypoxia with a quick recovery by incubating with 30% H_2_O_2_ in the last 1 h before further incubation with PBS and the probe (Figures 5m–o). The intensity of the fluorescence signal in the green channel recovered to be close to the control status. Accordingly, the hypoxia recovery with rational pulmonary ventilation might also be visualized by monitoring the GSH level. Therefore, the probe DSNBD-GSH might contribute to monitoring GSH in the cellular pulmonary ventilation model and inspire further optimized approaches.

## 4 Conclusion

In summary, to monitor GSH in the cellular pulmonary ventilation model, a fluorescent probe, DSNBD-GSH, was developed from the dichlorosalicylaldehyde raw material by introducing the nitrogen-containing aromatic rings and anchoring the NBD recognition group. Both the normoxia and hypoxia conditions were evaluated. In a solution containing DSNBD-GSH, the mimicked pulmonary ventilation did not obviously affect the absorption and fluorescence variation during the response towards GSH. When the excitation was set as 365 nm, the incubation with GSH caused the appearance of the fluorescence signal at 478 nm. The FQY value of the probe DSNBD-GSH was 0.11, while that of the detecting product DSNBD-FL was 0.69. The standard curve was constructed such that when the GSH concentration increased, the fluorescence intensity of DSNBD-GSH at 478 nm enhanced accordingly. There was a linear correlation when the GSH concentration was in the range of 0–800 μM, and the corresponding LOD value was 0.78 µM, which inferred a relatively high sensitivity. DSNBD-GSH suggested stable photophysical properties in various testing conditions of pH 5.0–11.0, 35 °C–45 °C. The reaction between DSNBD-GSH and GSH was absolutely completed within 15 min, which was a relatively rapid response. Furthermore, DSNBD-GSH ensured the high selectivity towards GSH and low cytotoxicity upon living pulmonary cells. Finally, DSNBD-GSH was available for monitoring the GSH level in the cellular pulmonary ventilation model. The hypoxia and recovery status inferred the efficacy of pulmonary ventilation, which was basically visualized by DSNBD-GSH. The introduction of the ESIPT mechanism with the substitutes on the backbone inspired further possibilities to regulate the capabilities of both the selectivity and signal production. More optimized fluorescent probes might improve the rationality of perioperative period strategies.

## Data Availability

The datasets presented in this study can be found in online repositories. The names of the repository/repositories and accession number(s) can be found in the article/Supplementary Material.
