# Supplementary material for: Developing a dichlorosalicylaldehyde-derived fluorescent probe for monitoring glutathione in a cellular pulmonary ventilation model
Source: Front Chem. 2025 Sep 30;13:1698116. doi: 10.3389/fchem.2025.1698116 (PMC12518257; doi:10.3389/fchem.2025.1698116)
Supplement: Supplementary file 1 [file DataSheet1.doc]

**Supplementary Materials**

**Developing** **a** **dichlorosalicylaldehyde-derived fluorescent probe for** **monitoring** **glutathione in** **cellular** **pulmonary ventilation model**

Xiao Zhou, Lei Zhang, Yuwen Lao, Bin Zhou, Zhongquan Zhu^*^

Department of Anesthesiology, Affiliated Jinhua Hospital, Zhejiang University School of Medicine, Jinhua, 321000, China.

*^*^Corresponding authors. E-mail: zhuzqzxyy@163.com.*

**Table of Contents**

NMR data 2

HRMS data 4

Absorbance and fluorescence variation of the responses in normoxia and hypoxia 5

Test on optical capabilities in various incubation temperature 5

Cell viability with different concentrations of the probe 5


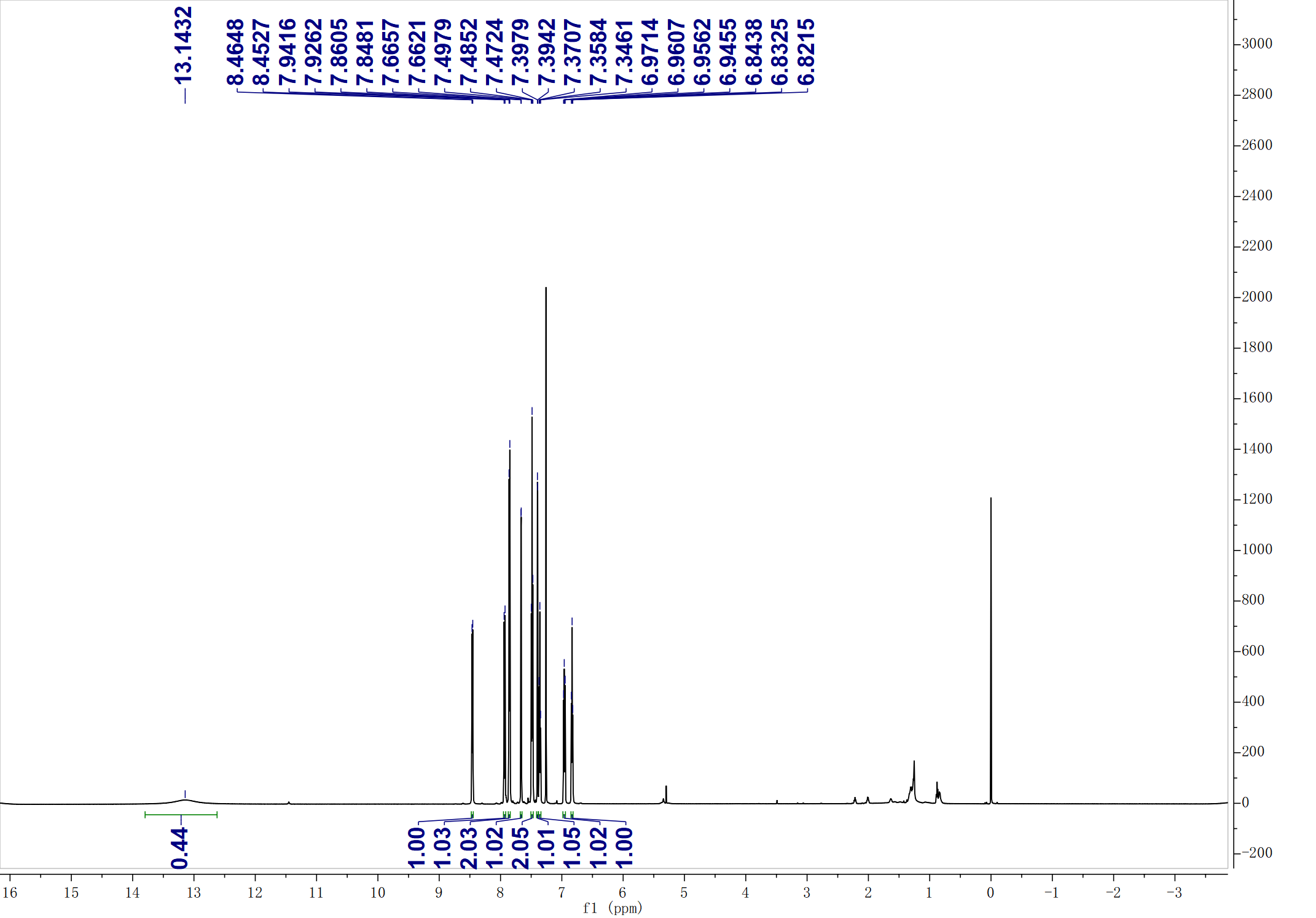


**Figure S1.** ^1^H NMR of the fluorophore **DSNBD-FL** (600 MHz, in CDCl_3_).


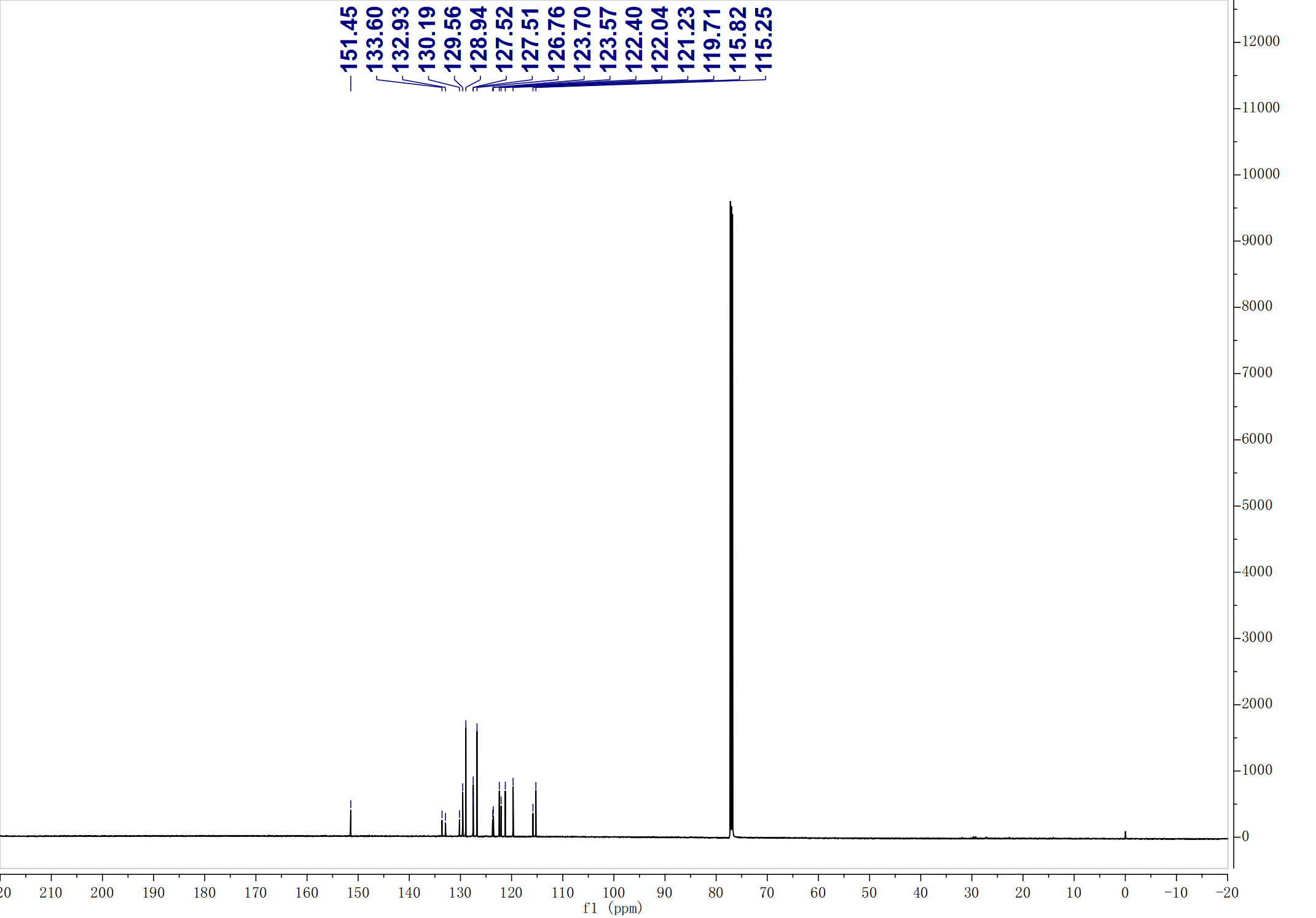


**Figure S2.** ^13^C NMR of the fluorophore **DSNBD-FL** (151 MHz, in CDCl_3_).


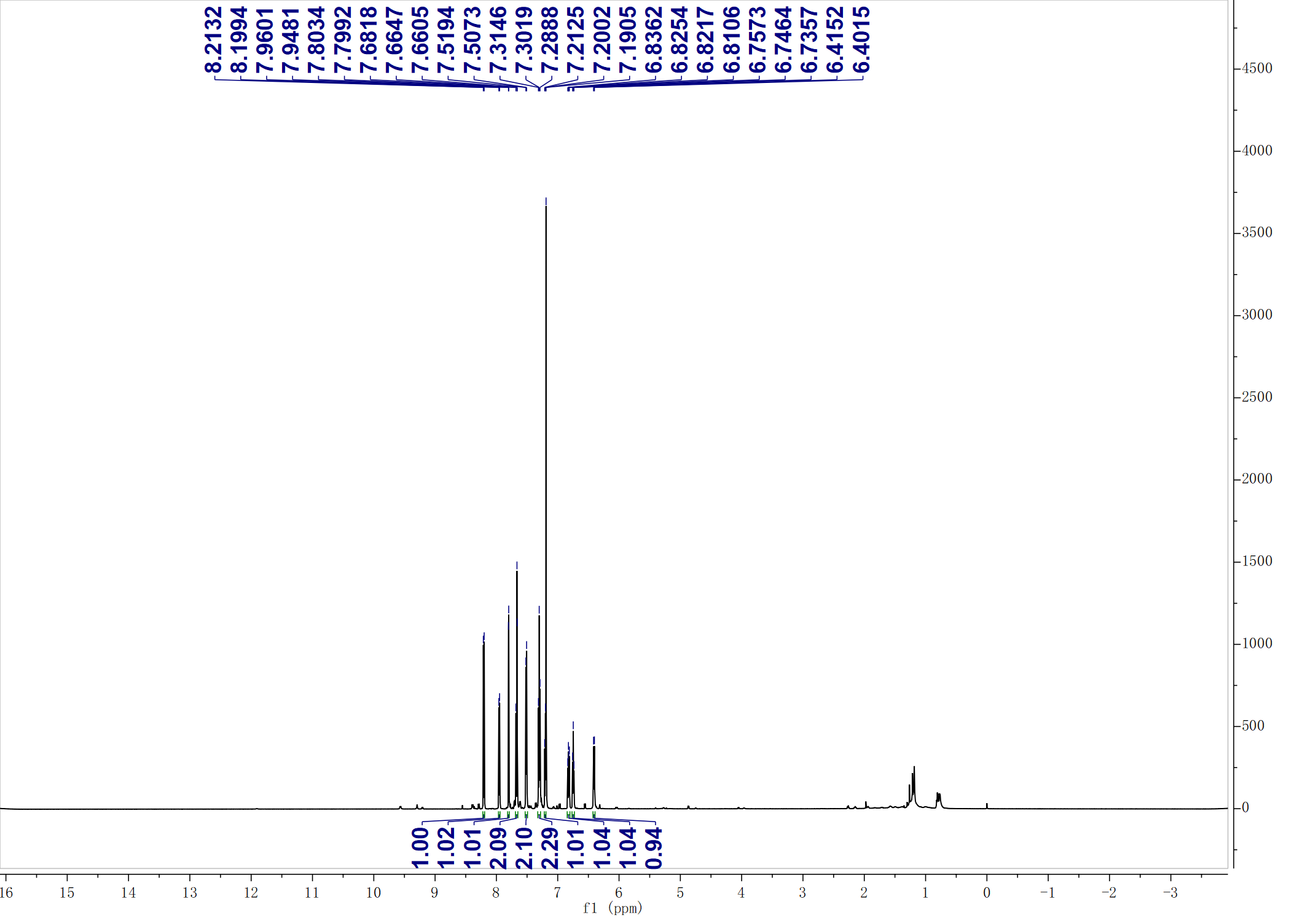


**Figure S3.** ^1^H NMR of the probe **DSNBD-GSH** (600 MHz, in CDCl_3_).


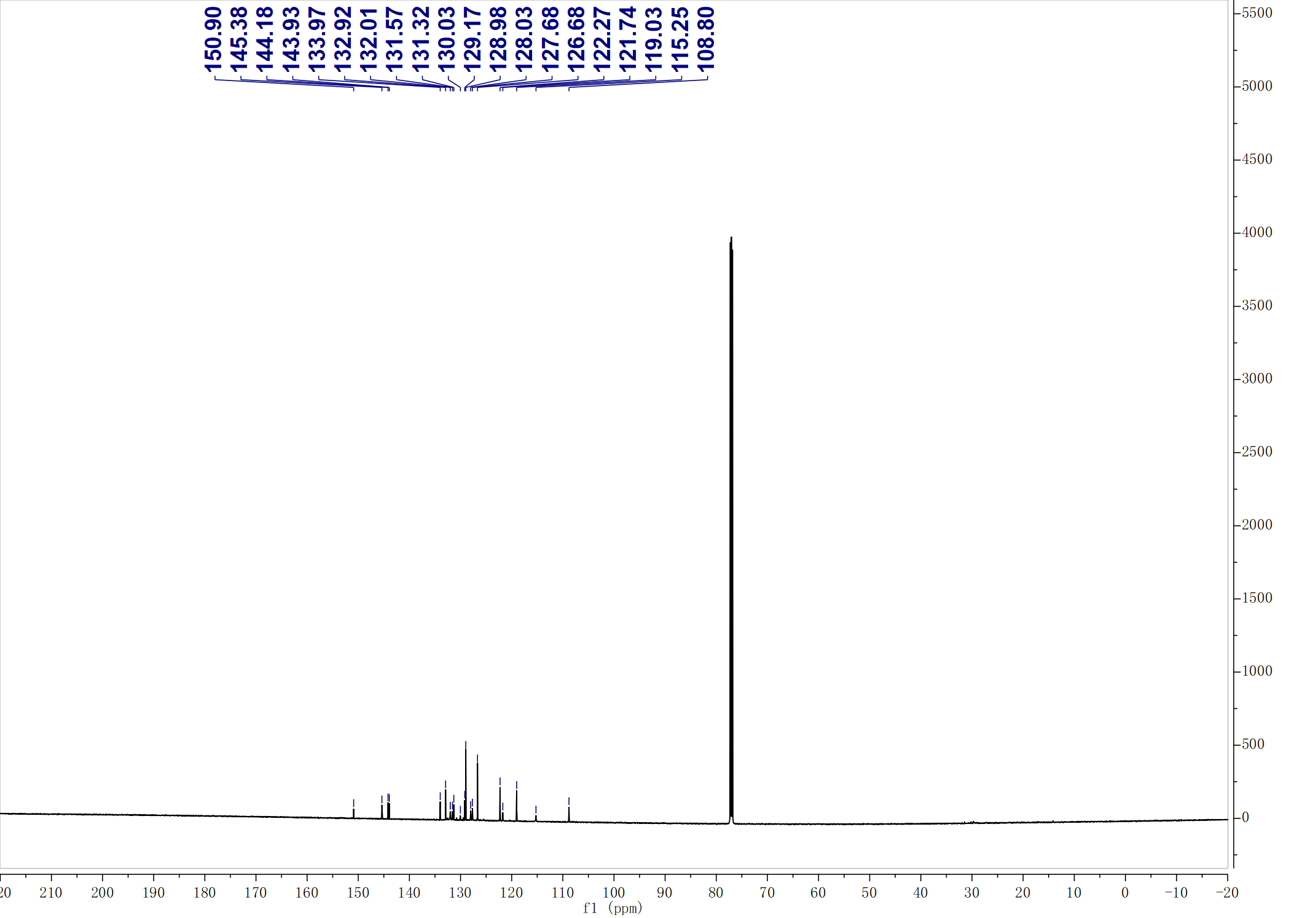


**Figure S4.** ^13^C NMR of the probe **DSNBD-GSH** (151 MHz, in CDCl_3_).


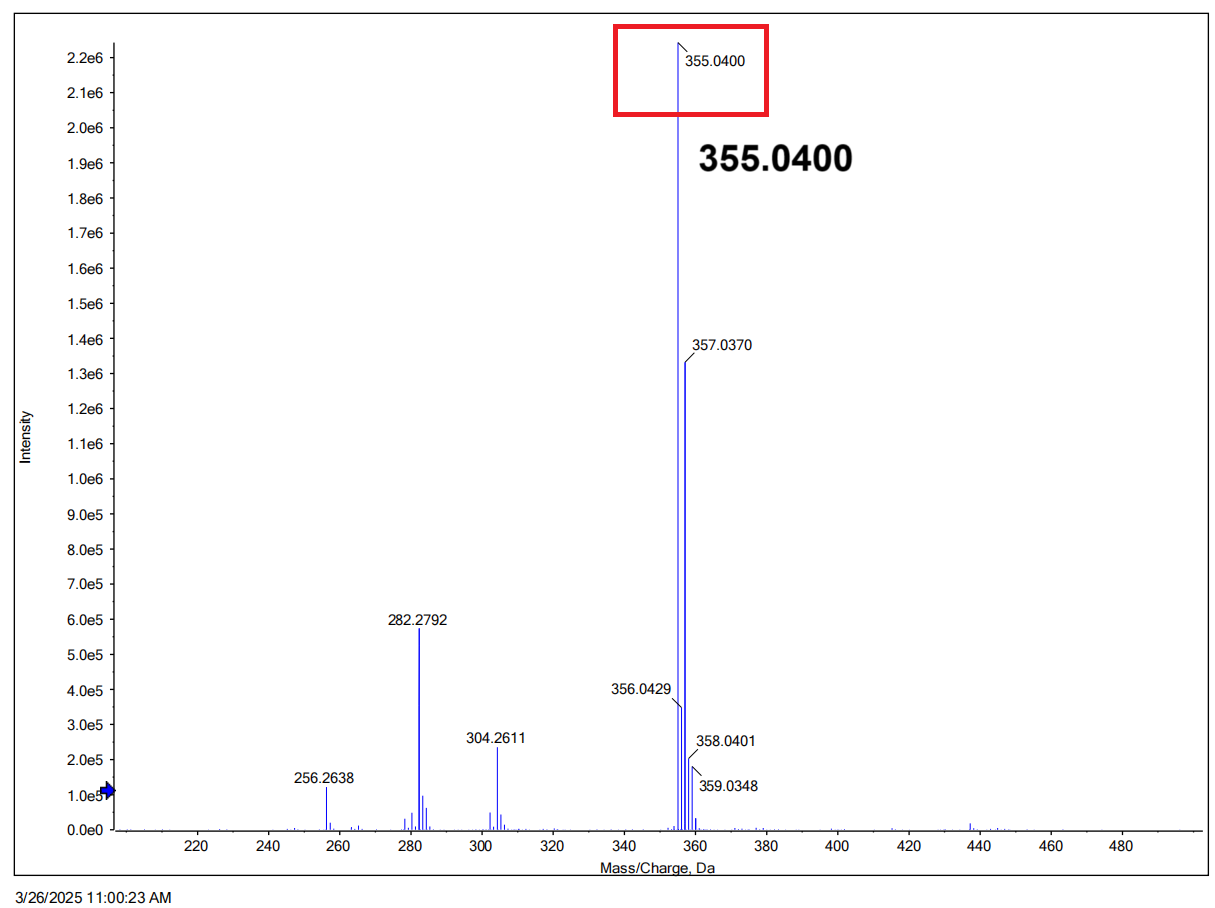


**Figure S5.** HRMS spectrum of the response product **DSNBD-FL** in methanol.


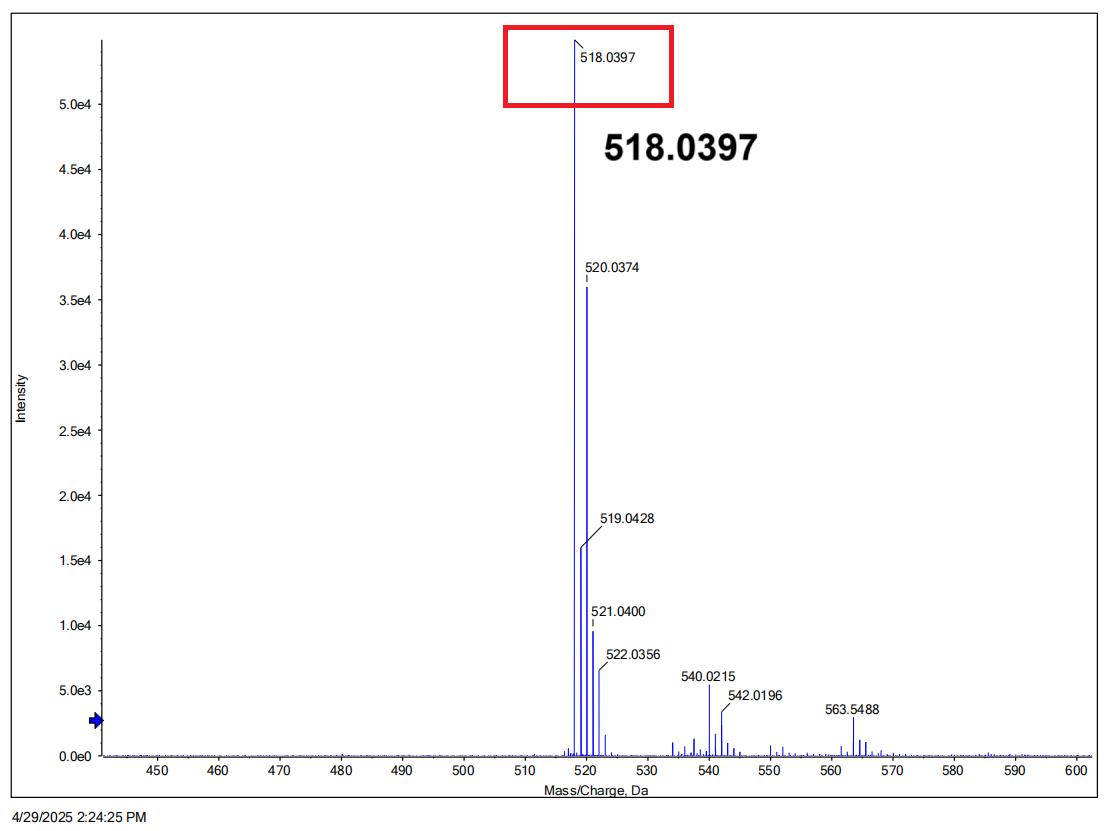


**Figure S6.** HRMS spectrum of the probe **DSNBD-GSH** in methanol.


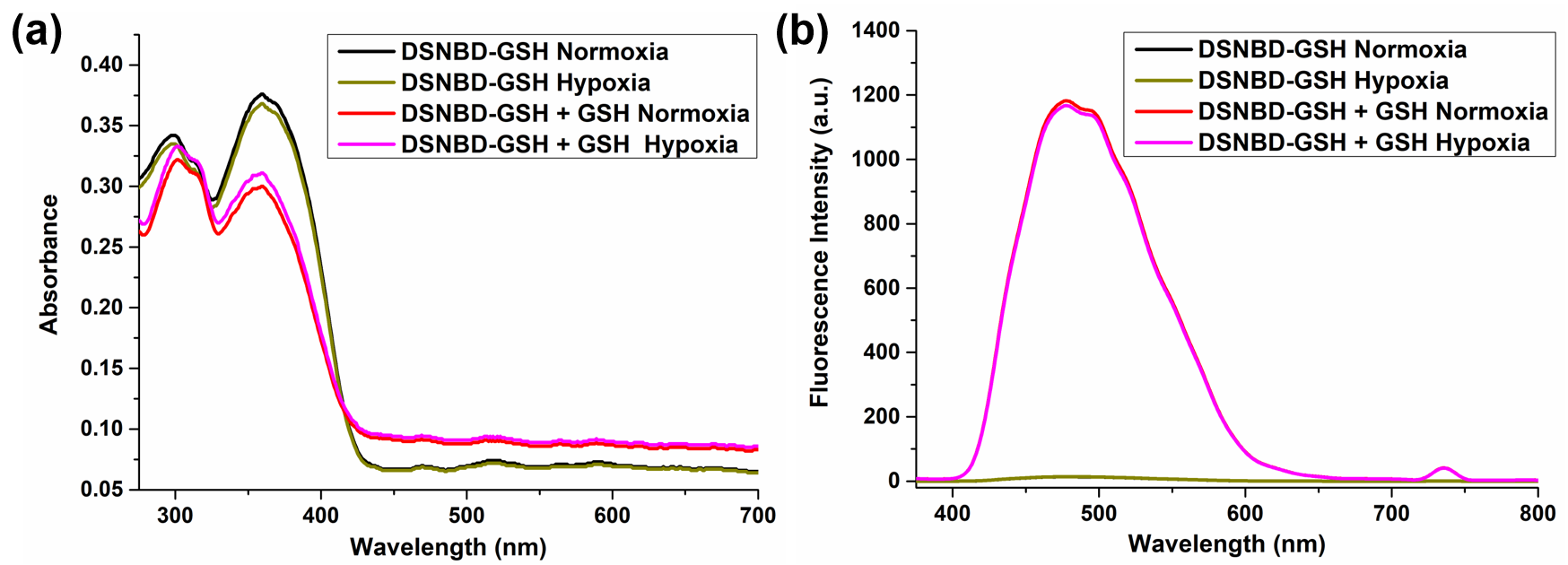


**Figure S7.** (a) The absorbance and (b) fluorescence spectra of the probe **DSNBD-GSH** (10 µM) in the absence and presence of GSH (1 mM). General testing conditions: 600 V, 5 nm * 5 nm, excitation wavelength 365 nm, pH 7.4, 20 min, 37 ℃.


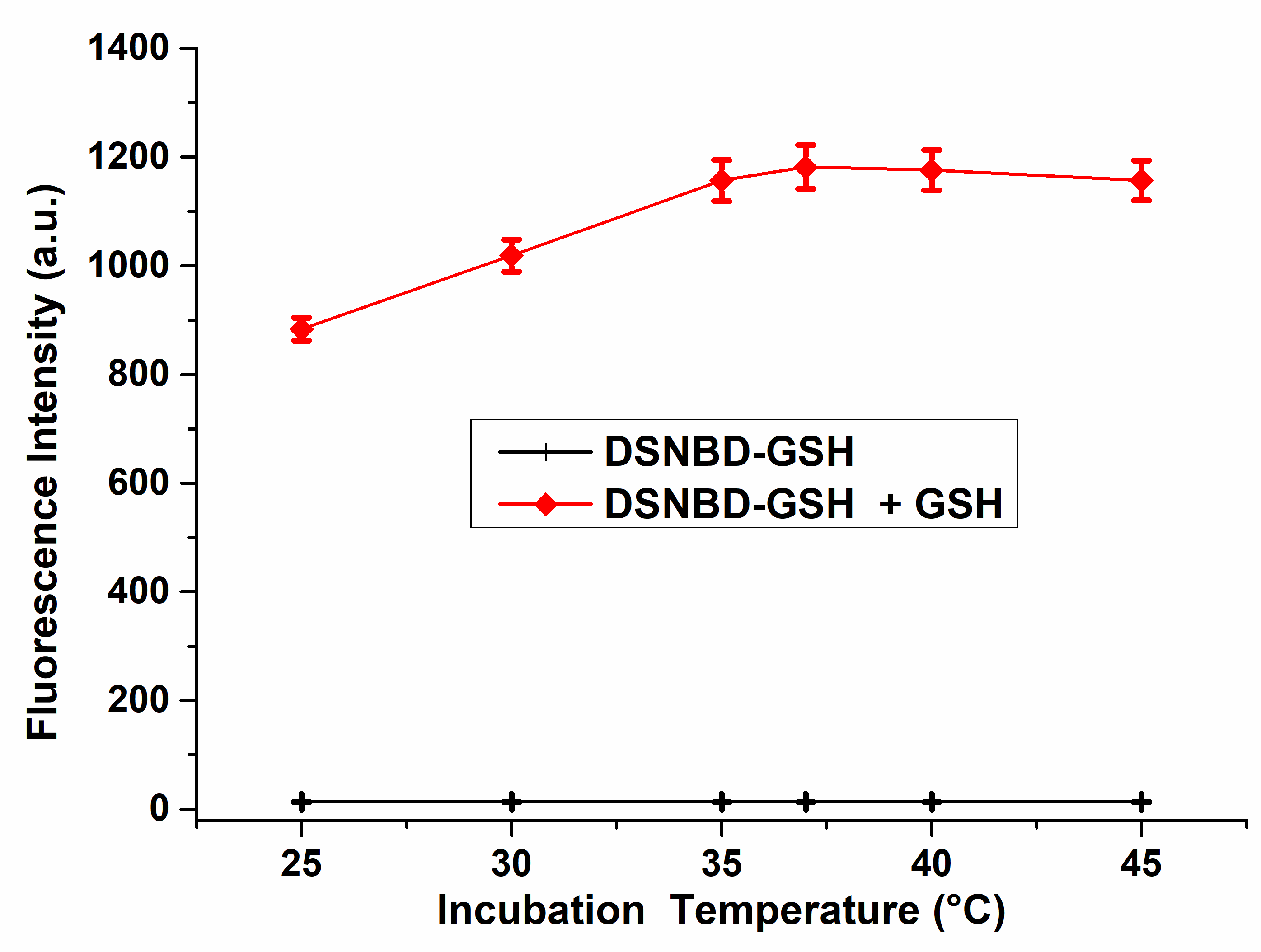


**Figure S8.** The fluorescence intensity variation of **DSNBD-GSH** (10 μM) with GSH (1 mM) in various incubation temperature (25-45 ℃). General testing conditions: 600 V, 5 nm * 5 nm, excitation wavelength 365 nm, pH 7.4, 20 min.


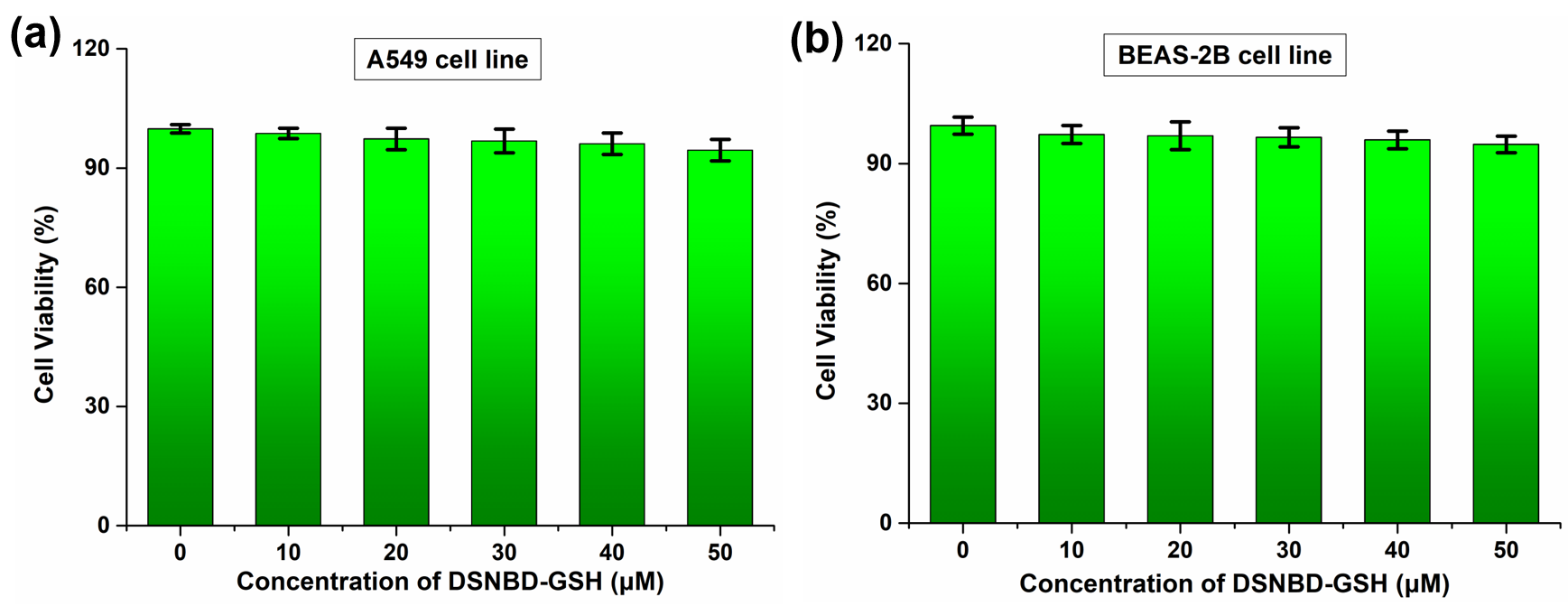


**Figure S9.** The cell viability of (a) A549 and (b) BEAS-2B cell lines after the incubation with different concentrations (0-50 μM) of **DSNBD-GSH** for 24 h at 37 ℃.
